# p68/DdX5 Supports β-Catenin & RNAP II during Androgen Receptor Mediated Transcription in Prostate Cancer

**DOI:** 10.1371/journal.pone.0054150

**Published:** 2013-01-17

**Authors:** Emma L. Clark, Christiana Hadjimichael, Richard Temperley, Amy Barnard, Frances V. Fuller-Pace, Craig N. Robson

**Affiliations:** 1 Northern Institute for Cancer Research, Newcastle University, Newcastle upon Tyne, United Kingdom; 2 Institute of Molecular Biology and Biotechnology, Heraklion, Crete, Greece; 3 Division of Cancer Research, Ninewells Hospital and Medical School, University of Dundee, Dundee, United Kingdom; Clermont Université, France

## Abstract

The DEAD box RNA helicase p68 (Ddx5) is an important androgen receptor (AR) transcriptional co-activator in prostate cancer (PCa) and is over-expressed in late stage disease. β-Catenin is a multifunctional protein with important structural and signalling functions which is up-regulated in PCa and similar to p68, interacts with the AR to co-activate expression of AR target genes. Importantly, p68 forms complexes with nuclear β-Catenin and promotes gene transcription in colon cancer indicating a functional interplay between these two proteins in cancer progression. In this study, we explore the relationship of p68 and β-Catenin in PCa to assess their potential co-operation in AR-dependent gene expression, which may be of importance in the development of castrate resistant prostate cancer (CRPCa). We use immunoprecipitation to demonstrate a novel interaction between p68 and β-Catenin in the nucleus of PCa cells, which is androgen dependent in LNCaP cells but androgen independent in a hormone refractory derivative of the same cell line (representative of the CRPCa disease type). Enhanced AR activity is seen in androgen-dependent luciferase reporter assays upon transient co-transfection of p68 and β-Catenin as an additive effect, and p68-depleted Chromatin-Immunoprecipitation (ChIP) showed a decrease in the recruitment of the AR and β-Catenin to androgen responsive promoter regions. In addition, we found p68 immunoprecipitated with the processive and non-processive form of RNA polymerase II (RNAP II) and show p68 recruited to elongating regions of the AR mediated *PSA* gene, suggesting a role for p68 in facilitating RNAP II transcription of AR mediated genes. These results suggest p68 is important in facilitating β-Catenin and AR transcriptional activity in PCa cells.

## Introduction

The onset and progression of prostate cancer (PCa) is driven by the transcriptional function of the androgen receptor (AR), and ablation of androgens is an effective strategy at early stages of the disease [1]. However, PCa can progress to a castrate resistant prostate cancer (CRPCa) phenotype that is currently untreatable [1], [2]. Aberrant activation of the AR is thought to play a prominent role in the development of CRPCa; a process postulated to be, in part, mediated via uncontrolled activation of co-activator proteins that facilitate the expression of AR responsive genes in a minimal hormone environment [3]. Understanding the molecular events by which progression to CRPCa occurs, may lead to the identification of novel targets and improve the survival of patients with disease.

β-Catenin is an integral component of the Wnt pathway, playing a role in signal transduction. The cytoplasmic stabilisation and nuclear accumulation of β-Catenin is the ‘hallmark’ of the activation of the Wnt signalling pathway (see reviews [4], [5]). In prostate cells, β-Catenin is found to be associated with liganded AR and act as an AR co-activator enhancing both Wnt and androgen responsive gene transcription (reviewed in [6]–[8]). Activated AR is able to shuttle β-Catenin into the nucleus and enhance AR transcription, indicating a ligand dependent interaction [9]. However, xenografts harvested from castrate resistant mice also demonstrated increased co-localisation and interaction of AR and β-Catenin [10]. In LNCaP PCa cells in the absence of androgens, H2-relaxin mediated phosphorylation of Akt and GSK-3β caused the stabilised cytoplasmic accumulation of β-Catenin which subsequently bound to the AR and translocated into the nucleus, suggesting that the presence of androgens is not essential for the interaction between AR and β-Catenin under certain conditions [11]. Interestingly, co-localization and interaction of AR and β-Catenin was not seen in tumours harvested from non-castrated mice, suggesting that this interaction is specific to the progression of PCa to CRPCa and warrants further investigation. Direct evidence of β-Catenin as part of the AR transcriptional complex has been demonstrated through Chromatin Immunoprecipitation (ChIP) studies, which show β-Catenin recruited to the promoter regions of both androgen and Wnt responsive genes in the presence and absence of androgens [11], [12]. Further evidence suggests that the growth of metastatic prostate tumour cells in the bone is via androgen mediated Wnt activation [13], and increased nuclear β-Catenin levels have been correlated with prostate cancer disease progression [14], [15]. In addition, reduction or loss of E-cadherin which normally sequesters β-Catenin at the plasma membrane is postulated to increase levels of cellular β-Catenin and promote AR activity [7]. Collectively, the data suggest an important role for β-Catenin in the progression of PCa to the CRPCa phenotype. However, it is clear that the precise mechanisms by which β-Catenin mediates AR transcriptional activity and growth of CRPCa in the absence of androgens, deserves further investigation.

The RNA helicase p68 (Ddx5) is a growth- and developmentally- regulated prototypic member of the DEAD box family of helicases. p68 functions in many cellular processes commonly dysregulated in cancer including processing of pre-mRNA and alternative splicing, cell proliferation, microRNA processing (reviewed in [16], [17]), and ribosome biogenesis [18]. p68 is also known to interact with several components of the transcriptional complex and to co-activate various transcription factors such as tumour suppressor p53, Estrogen Receptor α (ERα) and β-Catenin (reviewed in [16], [19], [20]). We have previously demonstrated p68 over-expressed in PCa and functioning as a co-activator of the AR [21]. In addition to prostate tumours, p68 is over-expressed in many other cancer cell types such as colon and breast, suggesting that p68 acts as a potential tumour promoter ( [22] and reviewed in [17]). p68 and the highly homologous protein p72 (Ddx17), are over-expressed and form complexes with β-Catenin in the nucleus of colon cancer cells to activate gene transcription and promote cell proliferation [22]–[24]. Tyrosine 593-phosphorylated p68 also associated with β-Catenin in the cytoplasm of colon cancer cells, where it promoted β-Catenin nuclear translocation via a RanGTPase Wnt-independent pathway through interaction with β-Catenin and displacement of Axin [25], [26]. However, conflicting research found no evidence that p68 was required for nuclear translocation of β-Catenin in the same cell type [27], and tyrosine 593-phosphorylated p68 did not differ from wild-type in its ability to stimulate β-Catenin dependent transcription in another study [23].

In this article, we use immunoprecipitation, ChIP and luciferase reporter techniques in combination with siRNA oligo nucleotide knockdown, to explore the relationship of p68 with β-Catenin to understand the molecular mechanism by which they potentially mediate aberrant activation of the AR and growth of PCa cells, as part of the AR transcriptional complex.

## Results

### p68 and β-Catenin Interact in the Nucleus of PCa Cells

**Figure 1:**
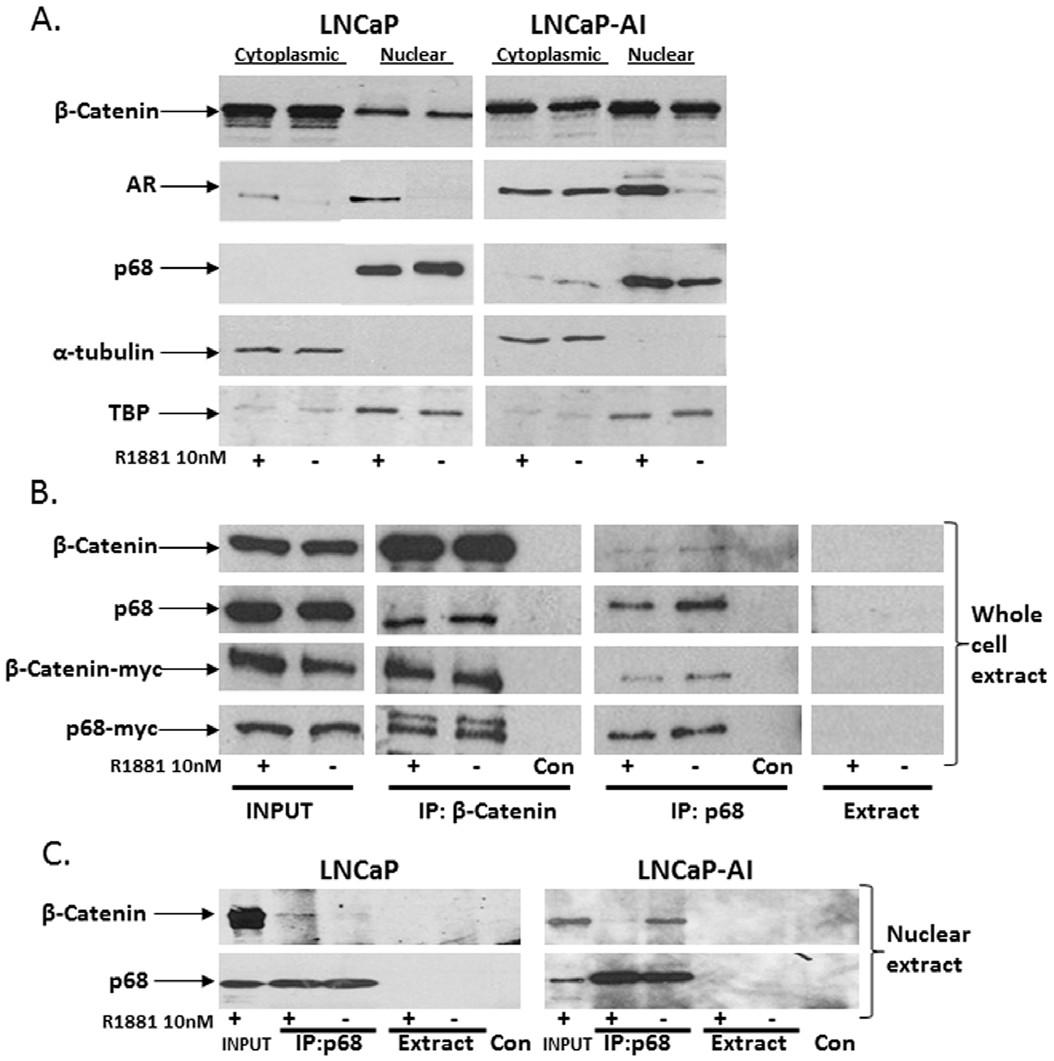
Localisation and interaction of p68 and β-Catenin in PCa cells. **A.** Cropped immuno-blot images show cytoplasmic and nuclear LNCaP and LNCaP-AI PCa cell lysates (+/− R1881 10 nM, 8 hours), probed sequentially with β-Catenin, AR, p68, α-tubulin and TATA binding (TBP) antibodies. **B.** Interaction of ectopic p68 and β-Catenin in COS-7 cells. Whole cell lysates of COS-7 cells transfected with pcDNA_3_-p68-myc and pCS^3+^-Myc_6_-β-Catenin constructs (+/− R1881 10 nM, 8 hours), were immunoprecipitated with β-Catenin and p68 antibody respectively. Cropped immuno-blots were probed sequentially with β-Catenin, p68 and myc antibody.**C**. Interaction of endogenous p68 and β-Catenin in the nucleus of LNCaP and LNCaP-AI PCa cells in the presence and absence of androgens. Cropped immuno-blot images of LNCaP and LNCaP-AI nuclear lysates, immunoprecipitated with p68 antibody (+/− R1881 10 nM, 8 hours), and probed sequentially with β-Catenin and p68. Extract samples contain either whole cell or nuclear lysate and protein G sepharose with no antibody present. Control (Con) samples contain antibody and protein G sepharose in extraction buffer only.

Given that the androgen receptor (AR) associates independently with β-Catenin and p68 in PCa cells [21], [28], and p68 and β-Catenin interact in colon cancer cells [23]. We speculate a possible three-way protein interaction between the AR, β-Catenin and p68 in PCa cells. Ligand free AR is sequestered within the cytoplasm of PCa cells and upon hormone binding moves predominantly into the nucleus [29]. Previously, we found p68 to be a nuclear protein in PCa cells whose localisation was unaltered by androgen treatment [21]. However, p68 has been found in the cytoplasm of colon cancer cells where it associates with β-Catenin [23], [25], and β-Catenin has been shown to interact with the AR in the cytoplasm of PCa cells and move into the nucleus in the presence and absence of androgens [9]–[11]. In light of these findings, we sought to confirm the localisation of β-Catenin and p68 in the LNCaP and the hormone refractory LNCaP-AI PCa cell line (see materials and methods). As expected in response to R1881 treatment (10 nM), AR translocated into the nucleus in both LNCaP and LNCaP-AI cells (Figure 1A). Androgen treatment did not significantly alter the nuclear localisation of p68 in either LNCaP or LNCaP-AI cells and similarly, the localisation of β-Catenin was unchanged upon R1881 treatment. Proportionally less β-Catenin is found in the nucleus compared to the cytoplasm of LNCaP cells, with proportionally higher in the LNCaP-AI cell type. This reflects previous findings that demonstrated AR constitutively shuttles β-Catenin into the nucleus of LNCaP-AI cells as an adaptation to androgen independent conditions [10], [12].

Co-immunoprecipitation of HCT-116 cell lysates with β-Catenin antibody identified an endogenous p68 interaction with β-Catenin in whole lysates of colon cancer cells [23]. Further analysis using Myc-tagged truncations of p68 demonstrated that the COOH terminus of p68 was unable to interact with β-Catenin, and the interaction was through the helicase (N-terminal) domain of p68. Following our immuno-blot findings in Figure 1A where both β-Catenin and p68 were found in the nucleus of LNCaP and LNCaP-AI cells in the presence and absence of androgens, we investigated whether p68 and β-Catenin directly interacted in the nucleus of PCa cells. Figure 1B shows ectopic co-immunoprecipitation of over-expressed myc-tagged p68 and β-Catenin fusion proteins in the androgen receptor negative COS-7 cell line, in both the presence and absence of R1881 (10 nM). Demonstrating that under *in-vitro* conditions, p68 and β-Catenin are able to directly bind independently of AR and androgens. (N.B. the higher band in the β-Catenin immunoprecipitation blot is a non-specific band detected by the myc antibody). However, endogenous protein immunoprecipitated from nuclear LNCaP extracts showed the β-Catenin-p68 interaction was facilitated in the presence of androgens (R1881, 10 nM), but conversely in the LNCaP-AI cell line the interaction was facilitated in the absence of androgens (R1881, 10 nM) (Figure 1C). (N.B. the reverse immunoprecipitation using β-Catenin antibody to pull down p68 protein failed to show an interaction between the two proteins in either the LNCaP or LNCaP-AI cell type, despite numerous attempts with various antibodies to β-Catenin). We also found no endogenous interaction between p68 and β-Catenin in the AR negative PC3 cell line (see Figure S1 in supporting information), indicating that the presence of a functional AR is possibly important for an endogenous interaction of p68 and β-Catenin in PCa cells [21], [28].

### p68 Interacts with RNAP II and is Recruited to Functional Regions of the *PSA* Gene

**Figure 2:**
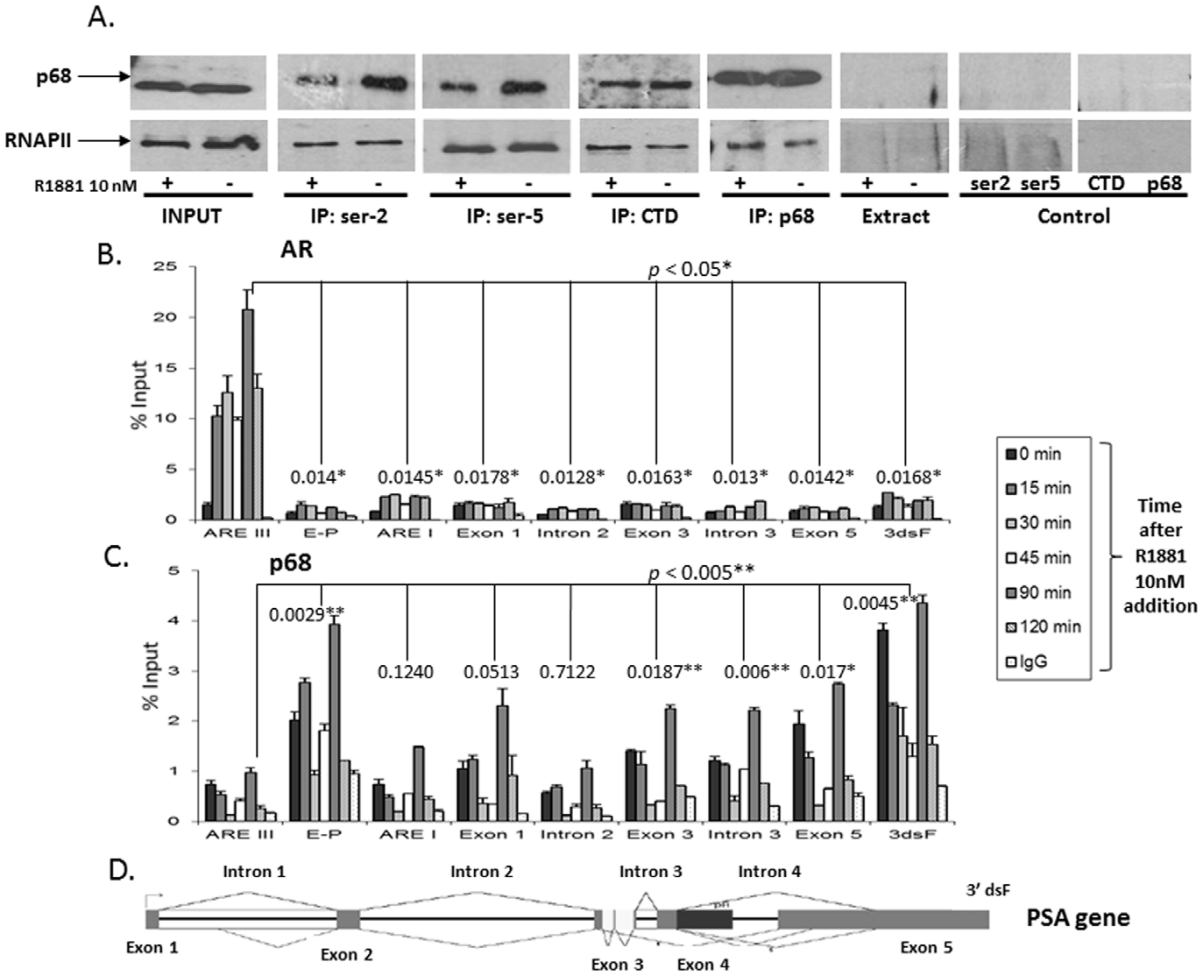
p68 interacts with RNAPII in PCa cells and occupies many regions of the *PSA* gene. **A.** Cropped immuno-blot images of LNCaP nuclear lysates immunoprecipitated with RNAP II H5 (ser-2), RNAP II H14 (ser-5), RNAP II CTD mouse monoclonal and p68 antibody (+/− R1881 10 nM, 8 hours), probed sequentially with p68 and RNAP II antibody. Extract samples contain nuclear cell lysate and protein G sepharose with no antibody present. Control samples contain antibody and protein G sepharose in nuclear extraction buffer only. **B.** Recruitment of AR and **C.** p68 to regions of the *PSA* gene. LNCaP cells were treated with 10 nM R1881 and harvested at 0, 15, 30, 45, 90 & 120 minute time points. Samples were immunoprecipitated with AR**,** p68 or control IgG antibodies and recovered material processed by ChIP assay. QPCR data are representative of n = 3 independent ChIP assays normalised to input levels (+/− SE). (N.B. QPCR primers could not be optimised to all exonic and intronic regions of the *PSA* gene). The independent paired sample *t* test was used to compare enrichment in recruitment between different *PSA* regions and show significance. **D.** Diagram of *PSA* gene depicting exon/intron boundaries.

We have previously described p68 as functioning as an ‘adaptor’ or ‘coupling’ protein that may coordinate the tightly integrated processes of transcriptional initiation, elongation and mRNA splicing in AR-regulated gene expression [30]. Recruitment of p68 to endogenous AR responsive genes may facilitate spliceosome assembly and increase the rate of RNA polymerase II (RNAP II) elongation, affecting splice site recognition and promoting exon skipping in nascent transcripts. Due to the established roles β-Catenin and p68 play in the transcriptional initiation of AR regulated genes, we sought to expand on these findings and investigate the relationship of p68 with RNAP II in PCa cells. We found p68 immunoprecipitated with both endogenous processive (phosphorylation at ser-2) and non-processive (phosphorylation at ser-5) forms of RNAP II in PCa cells, in both the presence and absence of androgens (Figure 2A R1881, 10 nM treatment). Indicating a possible role for p68 activity (in addition to AR co-activator function), during the elongation stages of AR regulated transcription. We previously demonstrated by Chromatin-Immunoprecipitation (ChIP) and QPCR analysis over a 100 minute androgen treatment (R1881, 10 nM) time course, a co-recruitment of p68 with the AR at the androgen responsive promoter and enhancer regions of the AR regulated *PSA* gene [21]. Expanding on these findings, we optimised QPCR primers to other areas of the *PSA* gene (in-between the enhancer and promoter region (E-P), exon 1, intron 2, exon 3, intron 3, exon 5 and 3′dsF regions), to establish whether p68 was recruited to sites other than transcriptional initiation. (N.B. The primer location and sequence information for these regions can be found in Table S1 in supporting information. It was not possible to optimise QPCR primers to intron 1, exon 2, intron 4 or exon 4 regions of the *PSA* gene). Figure 2B shows significant (*p*<0.05*) differential enrichment of AR upon R1881 treatment (10 nM) at set time points (0, 15, 30, 45, 90 and 120 minutes) to the ARE III region of the *PSA* gene, as demonstrated previously [31]. Enrichment of AR at other regions was not seen. Similarly, figure 2C shows significant (*p*<0.005**) cyclical dissociation-association recruitment of p68 upon R1881 treatment (10 nM) to the ARE III region, although recruitment was not limited to this region as E-P, Exon 3, Intron 3, Exon 5 and 3′dsF regions also showed significant enrichment. Recruitment did not reach significance at ARE I, Exon 1 and Intron 2 regions. These findings imply p68 is associated with both transcriptional initiation and elongation forms of RNAP II and enriched not only at the transcriptional start sites of a AR regulated gene but at exonic, intronic and 3′dsF regions, indicating a possible function for p68 in facilitating the processing of AR gene transcription by RNAP II.

### p68 Function is Required for Recruitment of AR and β-Catenin to the Promoter Regions of Androgen Responsive Genes

**Figure 3:**
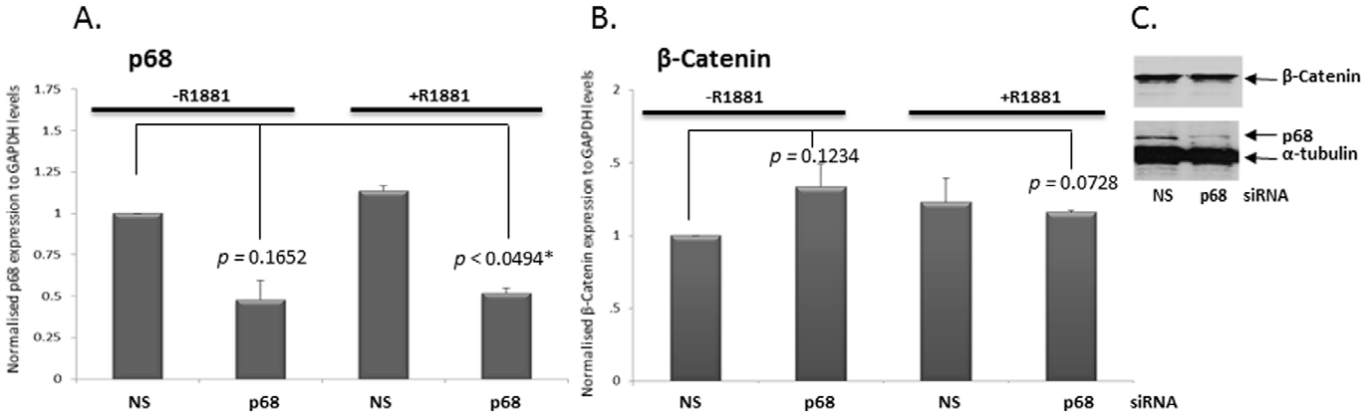
Knockdown of p68 does not alter β-Catenin mRNA or protein expression. mRNA expression levels of p68 **A.** and β-Catenin **B.** in control (NS) and p68 siRNA-transfected LNCaP cells (+/− R1881 10 nM, 16 hours). QPCR data was normalised to GAPDH levels and fold change calculated relative to control (NS) (-R1881) mRNA levels (set as 1). The independent sample *t* test was used to compare differences in expression levels and show significance. **C.** Cropped immuno-blot images of lysates from LNCaP cells treated with 10 nM R1881 (16 hours) and transfected with control (NS) and p68 siRNA. Blots probed sequentially with β-Catenin, p68 and α-tubulin antibodies.

**Figure 4:**
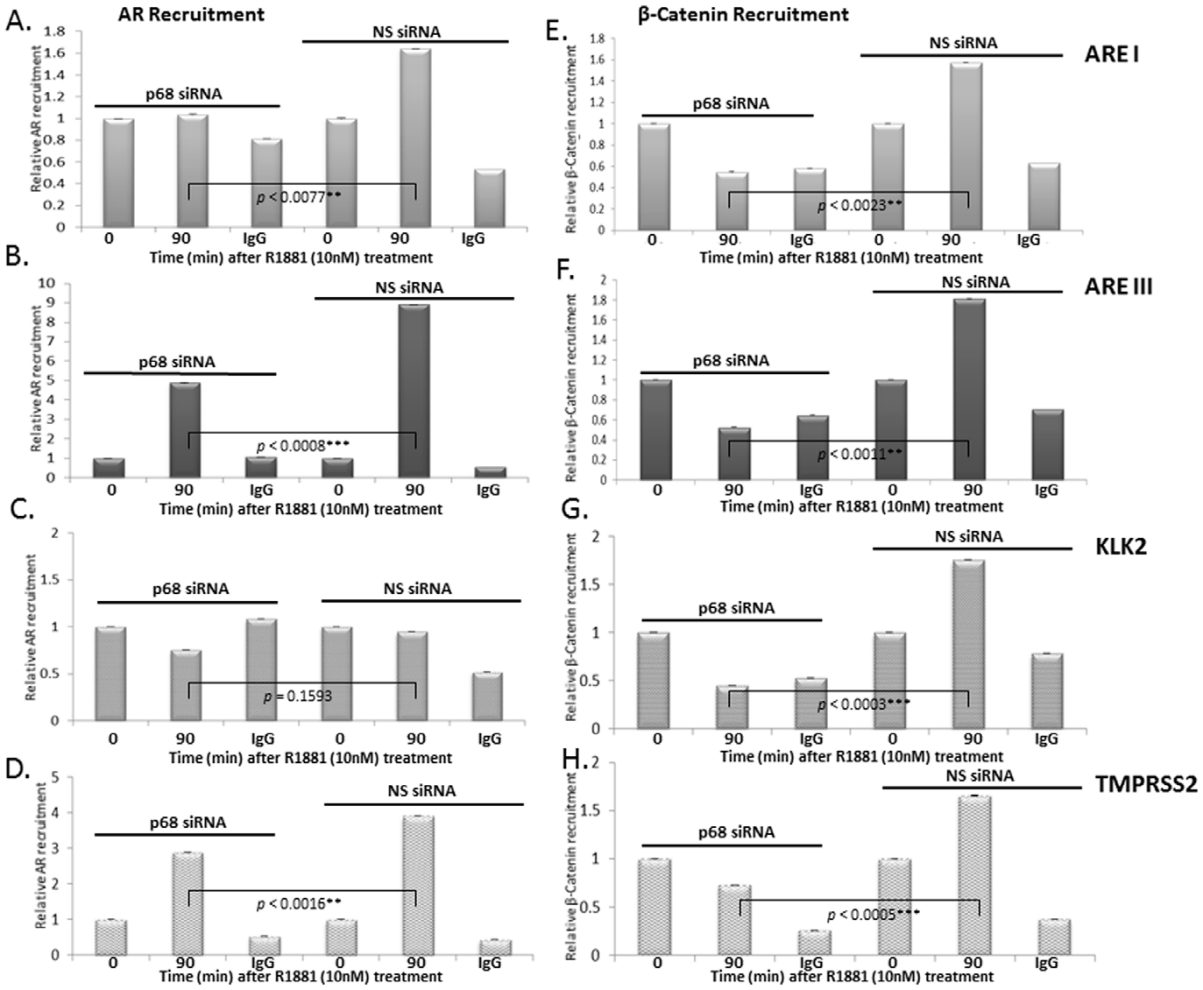
Depletion of p68 reduces AR and β-Catenin recruitment to promoter regions of androgen responsive genes. LNCaP cells transfected with p68 or control (NS) siRNA, treated with 10 nM R1881 for 90 minutes and immunoprecipitated with either AR **A. B.** **C. & D.** or β-Catenin **E. F. G. & H.** antibodies (including a IgG control antibody). Recovered material was processed by ChIP assay and recruitment to *PSA* ARE I (A & E), ARE III (B & F), *KLK2* (C & G) & *TMPRSS2* (D & H) promoter regions assessed relative to 0 minute time point. p68 depletion in LNCaP cells showed reduced AR & β-Catenin recruitment after treatment with 10 nM R1881 for 90 minutes to all regions assessed compared to control (NS) siRNA cells. Results shown represent n = 3 independent experiments (+/− SD). The independent sample *t* test was used to compare differences in expression levels and show significance.

We have previously shown a decrease in mRNA and protein levels of the AR and the androgen responsive *PSA* gene upon p68 knockdown by siRNA [21]. Consistent with the ChIP findings described above, β-Catenin is recruited to the promoter and enhancer regions of androgen responsive and Wnt signalling target genes in both the presence and absence of androgens [11], [12]. In light of these findings, we performed ChIP experiments in LNCaP cells depleted of p68 by siRNA to establish whether p68 function was required for AR and β-Catenin recruitment to the promoter regions of androgen responsive genes. p68 targeted siRNA knockdown expression (attenuation of p68 mRNA ∼50% *p*<0.0494* and protein∼90% at 72 hrs post transfection), did not significantly alter β-Catenin mRNA or protein expression levels compared to control (non-silencing, NS) siRNA in LNCaPs cells, in the presence or absence of R1881 (Figure 3A, B and C respectively). Androgen stimulation facilitated a modest (0.8 fold) recruitment of AR to the ARE I region of the *PSA* promoter in control (NS) siRNA transfected LNCaP cells as expected (Figure 4A), which was significantly attenuated to control levels in p68 depleted cells (*p*<0.0077**). Similarly, androgen stimulation increased AR recruitment to the ARE III enhancer region of the *PSA* gene 9 fold in control (NS) siRNA transfected cells (Figure 4B), whilst in p68 depleted cells, recruitment to the same region was reduced by 4 fold (*p*<0.0008***). A similar pattern of attenuation in AR recruitment was also seen at *KLK2* (0.25 fold, *p* = 0.1593 Figure 4C) and *TMPRSS2* (1 fold, *p*<0.0016** Figure 4D) promoter regions in p68 targeted siRNA depleted cells upon R1881 treatment, although this did not reach significance and androgen stimulation did not show enrichment of AR recruitment at the *KLK2* promoter. However, we have seen recruitment of the AR to the *KLK2* promoter at other time points (data not shown). Interestingly, a similar pattern in attenuation of β-Catenin recruitment upon p68 targeted siRNA knockdown was also seen. Increased β-Catenin recruitment to both ARE I (0.6 fold) and ARE III (0.8 fold) regions was observed upon androgen stimulation in control (NS) siRNA transfected cells compared to non-treated cells (Figure 4E and 4F respectively), as expected. However, in p68 depleted cells, β-Catenin recruitment was significantly attenuated to below control (NS) siRNA levels at both regions (1.1 fold, *p*<0.0023** and 1.3 fold, p<0.0011** respectively). A similar pattern of β-Catenin de-recruitment was repeated at the *KLK2* and *TMPRSS2* promoter in p68 depleted cells (1.25 fold, *p*<0.0003*** Figure 4G, and 1 fold, *p*<0.0005*** Figure 4H respectively). Although it is of note that in contrast to AR recruitment, an increase in β-Catenin recruitment (1.75 fold) is seen at the *KLK2* promoter region at this androgen time point. We have reported a decrease in AR mRNA and protein levels upon p68 depletion in LNCaP cells previously [21], supporting the notion that p68 functions as an AR co-activator. Therefore, a reduction in the recruitment of AR to the promoter regions of androgen responsive genes in p68 depleted cells would be expected as the AR itself is an androgen responsive gene. However in p68 depleted cells, we found β-Catenin recruitment reduced to levels lower than non-treated cells at all promoter regions assessed (similar to IgG levels). Suggesting direct p68 interaction may be required to facilitate the loading of β-Catenin to transcriptionally active regions of androgen responsive genes. This data highlights the importance of p68 function in the recruitment of the co-activator β-Catenin and the AR to the promoter regions of androgen responsive genes.

### p68 and β-Catenin Additively Enhance the Transcriptional Activity of Androgen Receptor Regulated Genes

**Figure 5:**
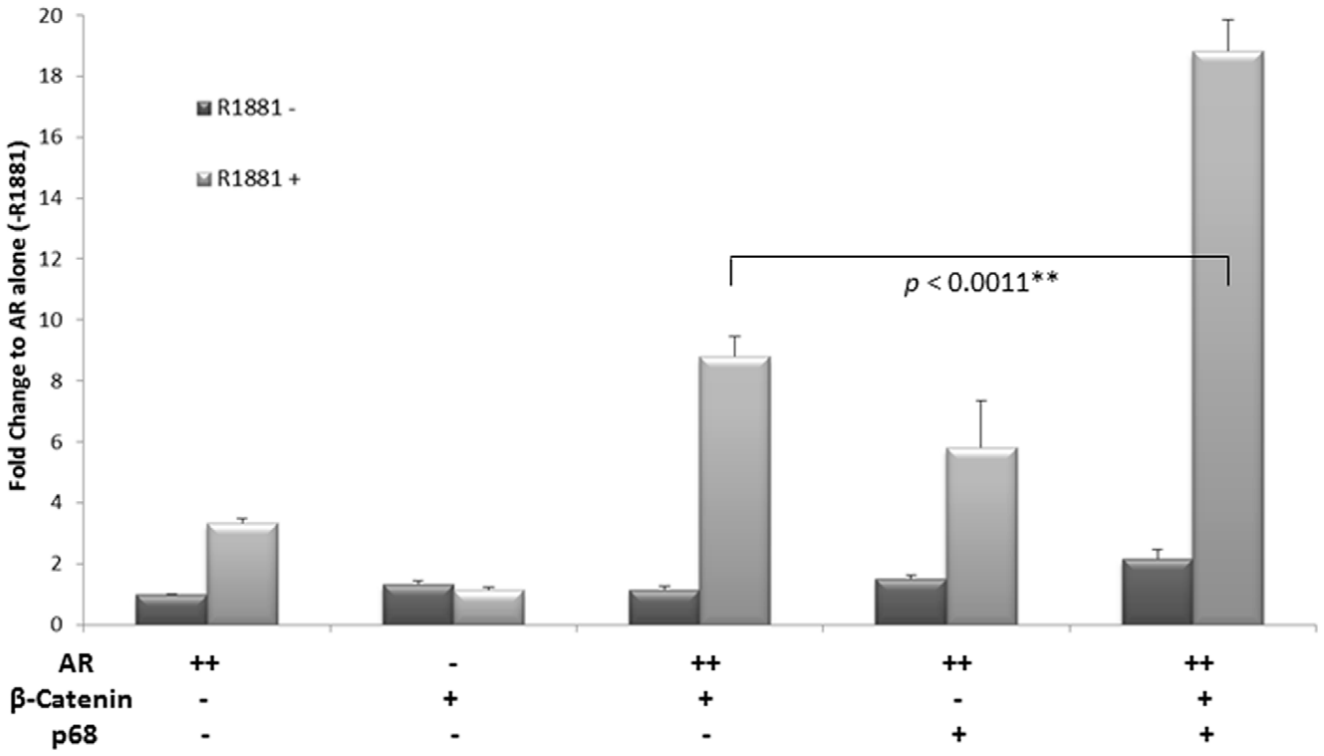
Over-expression of p68 additively enhances activity of β-Catenin mediated AR transcription. COS-7 cells transiently transfected in triplicate with p(ARE)_3_Luc reporter and PCMV-β-galactosidase plasmids together with mammalian expression vectors for AR, β-Catenin and p68 (+/−10 nM R1881). Luciferase activity was corrected for the corresponding β-galactosidase activity to give relative activity. The range of plasmid levels (+ and ++) corresponds to 50 and 100 ng respectively. Data shown relative to AR activity alone (−R1881) (set as 1), and representative of at least n = 3 luciferase assay experiments (+/− SE). The independent sample *t* test was used to compare differences in expression levels and show significance.

Given that p68 and β-Catenin interact in PCa cells and p68 facilitates the recruitment of β-Catenin to androgen responsive gene promoters. We next chose to investigate the combined effect of co-expression of p68 and β-Catenin on the transcriptional activity of the AR using androgen-dependent luciferase reporter assays in COS-7 cells. The AR-mediated p(ARE)_3_ Luc reporter was robustly stimulated 2 fold by the AR upon R1881 (10 nM) androgen treatment (Figure 5, compare light grey bar +R1881 and dark grey bar –R1881). Over-expression of β-Catenin showed negligible p(ARE)_3_ Luc reporter activity in the presence or absence of R1881 demonstrating that β-Catenin does not directly affect p(ARE)_3_ Luc reporter activity in the absence of AR. However, co-expression of AR and β-Catenin showed an 8 fold increase in p(ARE)_3_ Luc reporter activity upon R1881 treatment, confirming β-Catenin as a co-activator of the AR consistent with previous findings [28]. Similarly, co-expression of AR and p68 showed a 5 fold increase in p(ARE)_3_ Luc reporter activity following R1881 treatment, also confirming p68 as a co-activator of the AR consistent with previous findings. (N.B. p68 has been shown to not affect p(ARE)_3_ Luc reporter activity directly in the absence of AR [21]). However, co-expression of AR, β-Catenin and p68 constructs demonstrated a significant 18 fold (*p<*0.0011**) increase in p(ARE)_3_ Luc reporter activity upon R1881 treatment, 10 fold higher than AR and β-Catenin co-expression, demonstrating p68 has a significant additive effect on AR and β-Catenin transcriptional activity. This was also seen with another androgen regulated *PSA* promoter luciferase reporter (p(PSA) Luc), whereby co-expression of AR, β-Catenin and p68 constructs showed a significant 8 fold (*p*<0.0057**) increase in reporter activity, 5 fold higher than AR and β-Catenin co-expression, confirming the additive effect of p68 on β-Catenin and AR transcriptional activity (see supporting information, Figure S2). p68 has been reported to form heterodimers with the highly homologous helicase p72 (DdX17), and co-activate β-Catenin mediated gene expression previously [23]. We found that co-transfection of AR, β-Catenin and p72 constructs did not have a significant additive effect on p(ARE)_3_ Luc reporter activity compared to co-transfection of AR, β-Catenin and p68 constructs (see supporting information, Figure S3 *p* = 0.3646). This is similar to a previous report that found p72 was unable to enhance AR transcriptional activity of the p(ARE)_3_ Luc reporter [21], and suggests a specificity for p68 in the transcriptional activation of the AR in PCa. Collectively, these data suggest β-Catenin and p68 may work together as co-activators to enhance AR regulated transcription.

## Discussion

In this study, we describe a functional interaction between p68, β-Catenin and the AR in the nucleus of PCa cells. The AR has been shown to signal through the Wnt/β-Catenin pathway in PCa as an adaptation to castrate levels of androgens [12], and β-Catenin is known to interact with other co-activators of the AR [32]. Here we present immunoprecipitation, p68-depleted ChIP and *PSA* luciferase reporter data to confirm an interaction between p68, β-Catenin and the AR in PCa cells. We initially confirmed a direct *in-vitro* p68-β-Catenin interaction by transient transfection of constructs into the AR negative COS-7 cell line (which was not androgen dependent). However, upon further investigation we found under endogenous conditions the p68-β-Catenin interaction was facilitated in the presence of androgens in the LNCaP PCa cell line, but conversely in the hormone refractory (LNCaP-AI) derivative of this cell line (representative of the CRPCa disease type), the interaction was facilitated in the absence of androgens. This is similar to a finding of increased endogenous AR/β-Catenin complex formation in a castrate resistant PCa mouse xenografts model, where no interaction between AR and β-Catenin was detected in the presence of androgens [10]. This may suggest an adapted mechanism by which p68 and β-Catenin co-activators co-operatively maintain transcriptional activity of the AR (and androgen regulated genes), facilitating cell survival of the castrate-resistant (CRPCa) disease type. However, further work is required to substantiate the full molecular mechanism of this claim. Immuno-blot data confirmed the cellular localisation of p68 and β-Catenin was not influenced by hormone (we found p68 and β-Catenin in the nucleus of PCa cells in both the presence and absence of androgens), and *PSA* luciferase reporter data showed co-transfection of p68 and β-Catenin constructs had an additive effect on the transcriptional activity of AR in the presence of androgens. These data suggest that p68 and β-Catenin work together as co-activators to additively enhance the transcriptional activity of the AR which interestingly, may have implications in the progression of the CRPCa disease type.

We also showed using knockdown of p68 expression by siRNA oligonucleotide combined with ChIP, that p68 was required for optimal recruitment of the AR and β-Catenin to promoter regions of androgen regulated genes. Androgen stimulation facilitated recruitment of the AR to both ARE I and ARE III regions of the *PSA* promoter, and *KLK2* and *TMPRSS2* promoter regions in control (NS) siRNA transfected LNCaP cells, which was subsequently attenuated in p68 depleted cells. The observed β-Catenin recruitment to the same androgen regulated promoter regions was reduced in p68 depleted LNCaP cells upon androgen treatment, but to levels lower than non-treated cells (similar to the IgG control). This is an interesting finding as it suggests that a reduction in the recruitment of β-Catenin to the promoter regions of androgen regulated genes in p68 depleted cells is not just a consequence of less AR available for recruitment, and implies that p68 function is required to load β-Catenin to transcriptionally regulated regions of androgen responsive genes.

The role of p68 in alternative mRNA splicing is well documented (reviewed in [19], [21], [33]). We have previously speculated that p68 may function as an ‘adaptor’ or ‘coupling’ protein that coordinates the tightly integrated processes of transcription and RNA processing, facilitating cross-talk between transcription and RNA processing in AR-regulated genes, possibly by controlling the rate of transcriptional initiation/elongation of RNAP II [30]. RNAP II has two physiologically important phosphorylation sites at the C-terminus which are important for transition of the polymerase from a transcriptional initiation form (phosphorylation at ser-5), to the establishment of the elongation transcriptional complex form (phosphorylation at ser-2). In this study, we demonstrate using endogenous nuclear extracts of LNCaP PCa cells, that p68 interacts with both the processive (phosphorylation at ser-2) and non-processive (phosphorylation at ser-5) form of RNAP II, in the presence and absence of androgens. These findings directly link p68 to RNAP II during transcriptional initiation and elongation in PCa cells, suggesting that p68 (in addition to its role as an established AR transcriptional co-activator), could potentially facilitate the elongation of AR regulated genes. This is supported by ChIP data that provides evidence of p68 not only occupying promoter and enhancer regions of the AR regulated *PSA* gene, but also in-between the enhancer and promoter region and at exonic and intronic regions. Collectively, this suggests p68 has the potential to support RNAP II function at elongating regions of the *PSA* gene. ChIP recruitment data was gathered at set time points of androgen stimulation and recruitment to all areas of the *PSA* gene was seen in the absence of androgens. Immunoprecipitation data also showed p68 interacted with both forms of RNAP II in the absence of androgens. This suggests that p68 is possibly pre-loaded to transcriptionally active areas of the *PSA* gene ready for the start of transcription in a similar mode by which RNAP II is found ‘poised’ at transcriptionally active gene regions prior to transcriptional initiation [34], possibly facilitating potential ‘cross-talk’ between the AR and RNAP II. The notion that p68 is functioning as an adaptor, co-ordinating transcription and mRNA processing of AR regulated genes, is also consistent with the established role of p68 as a functional splicing factor [21], [35]. We also found association-dissociation recruitment of p68 at the 3′dsF region of the *PSA* gene suggesting a role for p68 in the termination and polyadenylation of AR mediated transcripts, consistent with a previous report that described p68 involved in transcriptional deactivation in *Drosophila* promoting mRNA transcript release [36]. During the preparation of this manuscript, Germann *et al.* published a manuscript which demonstrated p68 (Ddx5) and the highly homologous p72 (Ddx17) protein have a dual role in the control of transcription of the pro-migratory NFAT5 transcription factor (in addition to their role in facilitating the inclusion of NFAT5 exon 5), in breast cancer cells [37]. This work strengthens our findings and suggests p68 is possibly working as a ‘coupling’ factor to facilitate not only AR-regulated transcription in PCa, but other nuclear transcriptional factors in alternative cancer types. However, further work will be required to expand on these findings.

Collectively, the data presented in this manuscript has corroborated previous findings which identified p68 as an important co-activator of the AR, and expanded on these foundations to identify a novel role for p68 in facilitating the recruitment of not only the AR but the AR co-activator β-Catenin to androgen regulated genes. We demonstrate that p68 is not only integral to AR regulated transcription at the promoter level (transcriptional initiation), but also during elongation and transcriptional progression and may have the potential to influence the expression of AR regulated genes through direct interaction with RNAP II. Taken together, these data provide evidence that p68 is required for transcriptional regulation of AR mediated genes and is of importance in the recruitment of AR co-factors to the AR transcriptional complex. This may be of significance in the progression of the CRPCa disease type.

## Materials and Methods

### Cell Culture

All cells were grown at 37°C in 5% CO_2_. LNCaP and PC3 cells (American Type Culture Collection, ATCC, Manassas, VA), were cultured in RPMI-1640 and COS-7 cells (ATCC) in D-MEM media, both supplemented with 1% L-glutamine (Invitrogen) and 10% foetal calf serum (FCS) (Invitrogen). LNCaP-AI cells derived from the LNCaP cell line following continuous passaging in steroid depleted media for 8 months to gain androgen independence have been described previously [38], and were cultured in RPMI-1640 supplemented with 1% L-glutamine (Invitrogen) and 10% Dextran Charcoal Stripped FCS (Hyclone), to produce steroid-depleted media (SDM). Where indicated in figure legends, cells were treated with 10 nM of the synthetic androgen R1881 (Methyltrienolone).

### Plasmids, Antibodies and Drugs

The following plasmids have been described previously, p(ARE)_3_ Luc, PSA promoter Luc, pCMV-β-galactosidase, pcDNA_3_-AR, pcDNA_3_-p68, pcDNA_3_-p72 [21], [39]. The pCS^3+^-Myc_6_-β-Catenin and pSG5-AR constructs were kind gifts from Professor Ralf Janknecht (Mayo Clinic College of Medicine, USA) [23]. The following antibodies were used as indicated in figure legends; goat polyclonal p68 C-20 and mouse monoclonal β-Catenin 8E4 (Santa Cruz), mouse monoclonal p68 PAb204 and RNA polymerase II CTD4H8 (Millipore), mouse monoclonal AR (BD Pharmingen), rabbit polyclonal β-Catenin and mouse monoclonal TATA Binding Protein TBP (Abcam), mouse monoclonal RNA polymerase II CTD 8WG16, H5 (Ser2) and H14 (Ser5) (Covance), mouse monoclonal α-tubulin (Sigma) and mouse monoclonal Myc-Tag 9B11 (Cell Signalling). The p68 2906 rabbit polyclonal (raised against the C-terminal 15 residues of p68) has been described previously [40], [41].

### Luciferase Reporter Assays

Luciferase reporter assays were performed in COS-7 cells co-transfected in triplicate with 0.1 µg of p(ARE)_3_Luc or p(PSA)Luc, pcDNA_3_-AR or pSG5-AR and pCMV-β-galactosidase using Superfect (Qiagen), as described previously [31]. pcDNA_3_-p68, pcDNA_3_-p72 and pCS^3+^-Myc_6_-β-Catenin fusion constructs were co-transfected into cells as detailed in figure legends. The range of plasmid levels indicated in figures (+ and ++) corresponds to 50 and 100 ng respectively. Luciferase activity was corrected for corresponding β-galactosidase activity to give relative activity. All reporter assays are the mean of at least three independent experiments ± standard error.

### Cell Lysate Preparation and Co-Immunoprecipitation (IP)

LNCaP or LNCaP-AI cells were cultured in SDM for 48 hours prior to 10 nM R1881 treatment, (8 hours). Cells were nuclear extracted using CelLytic™ NuCLEAR™ Extraction kit (Sigma, UK) with an extraction buffer salt concentration of 330 mM in the presence of protease inhibitors (Roche, UK), and RNase A (100 µg/ml - Sigma). All immunoprecipitation experiments were performed as described previously [42].

### siRNA Knockdown and Quantitative Real-Time PCR (RT-QPCR)

LNCaP cells seeded in 6-well plates were reverse transfected with p68 or control (non silencing, NS) small interfering RNA (siRNA) (see [43], [44] for sequences), using lipofectamine RNAiMax (Invitrogen, according to the manufacturer’s instructions), and grown in SDM for 48 hours prior to treatment with 10 nM R1881 for a further 16 hours. Cells were harvested in either SDS-sample buffer to acquire protein for western blot analysis or TRIzol reagent (Invitrogen) followed by MMLV reverse transcription to study mRNA expression by RT-QPCR relative to GAPDH, as previously described [44]. β-Catenin (Forward AGGCTACTGTTGGATTGATTCGAA and Reverse CATGATTTGCGGGACAAAGG), p68 and GAPDH oligonucleotides (described previously [21]), were used to determine mRNA expression.

### Chromatin-Immunoprecipitation (ChIP) Assays

LNCaP cells seeded in 90mM dishes were reverse transfected with p68 or NS siRNA as described above and cultured in SDM for 48hours prior to 10 nM R1881 treatment for 90 minutes. ChIP was undertaken on siRNA treated cells using both AR and β-Catenin antibodies and an IgG antibody was included as a control, as described previously [21]. QPCR was subsequently performed on inputs and recovered material to assess the occupancy of AR and β-Catenin at the promoter ARE I and enhancer ARE III regions of the *PSA* gene, and the androgen responsive promoter regions of the *KLK2* and *TMPRSS2* genes (see Table S1 in supporting information for primer sequences). To assess p68 and AR occupancy at the promoter, coding and 3-UTR regions of the *PSA* gene, LNCaP cells were cultured in SDM for 48 hours prior to 10 nM R1881 treatment (at set time points as stipulated in the figure legend). The following regions were assessed for p68, AR and IgG (control antibody) occupancy: AREIII, AREI, in between the enhancer and promoter regions (E-P), Exon I, Intron 2, Exon 3, Intron 3, Exon 5 and the 3′dsF. The primer sequences for ARE III, ARE I and E-P have been described previously [42], [45]. All other QPCR primer sequences were optimised for this study and can be found in supporting information (Table S1), including primer position on the *PSA* gene. ChIP sonication products were optimised to robustly yield fragments of less than 500 bp to allow discrimination between all regions examined. ChIP results are representative of at least three independent experiments ± standard error. IgG control ChIP was performed on the longest androgen treatment set time point (120 min).

### Statistical Analysis

The independent sample *t* test was used with GraphPad Prism 6 software (GraphPad Software, Inc), to compare differences as stipulated in figure.

## Supporting Information

Figure S1 **Immunoprecipitation of endogenous p68 and β-Catenin in an AR negative PC3 cell line do not interact.** Cropped immuno-blot images of PC3 whole cell lysates, immunoprecipitated (IP) with either p68 or β-Catenin antibody and probed sequentially with β-Catenin and p68. Extract samples contain whole cell lysate and protein G sepharose with no antibody present, and Control (Con) samples contain antibody and protein G sepharose in extraction buffer only.
(TIF)

Figure S2 **Over-expression of p68 additively enhances the activity of β-Catenin mediated AR transcription by an AR mediated** ***PSA*** **promoter luciferase reporter.** COS-7 cells were transiently transfected in triplicate with 0.1 µg of p(PSA)Luc reporter, 0.1 µg pSG5-AR, 0.1 µg of pCMV-β-galactosidase and 0.05 µg of pcDNA3-p68 or pCS3+-Myc6-β-Catenin constructs (+10 nM R1881). Luciferase activity was corrected for the corresponding β-galactosidase activity to give relative activity. The range of plasmid levels (+ and ++) corresponds to 50 and 100ng respectively. Data is shown relative to AR activity alone (set as 1) and from at least three independent luciferase assay experiments (+/− SE).
(TIF)

Figure S3 **Over-expression of p72 does not enhance the activity of β-Catenin mediated AR transcription.** COS-7 cells were transiently transfected in triplicate with 0.1 µg of p(ARE)3 luciferase reporter, 0.1 µg pcDNA3-AR, 0.1 µg of pCMV-β-galactosidase and 0.05 µg of pcDNA3-p72, p68 or pCS3+-Myc6-β-Catenin constructs (+10 nM R1881). Luciferase activity was corrected for the corresponding β-galactosidase activity to give a relative activity. The range of plasmid levels (+ and ++) corresponds to 50 and 100 ng respectively. Data is shown relative to AR and β-Catenin activity alone (set as 1) and from at least three independent luciferase assay experiments (+/− SE).
(TIF)

Table S1 **Sequences for primers used in ChIP experiments and location of primers on the** ***PSA*** **gene.**
(TIF)
