# Supplementary figures and images for: p68/DdX5 Supports β-Catenin & RNAP II during Androgen Receptor Mediated Transcription in Prostate Cancer
Source: PLoS One. 2013 Jan 17;8(1):e54150. doi: 10.1371/journal.pone.0054150 (PMC3547877; doi:10.1371/journal.pone.0054150)

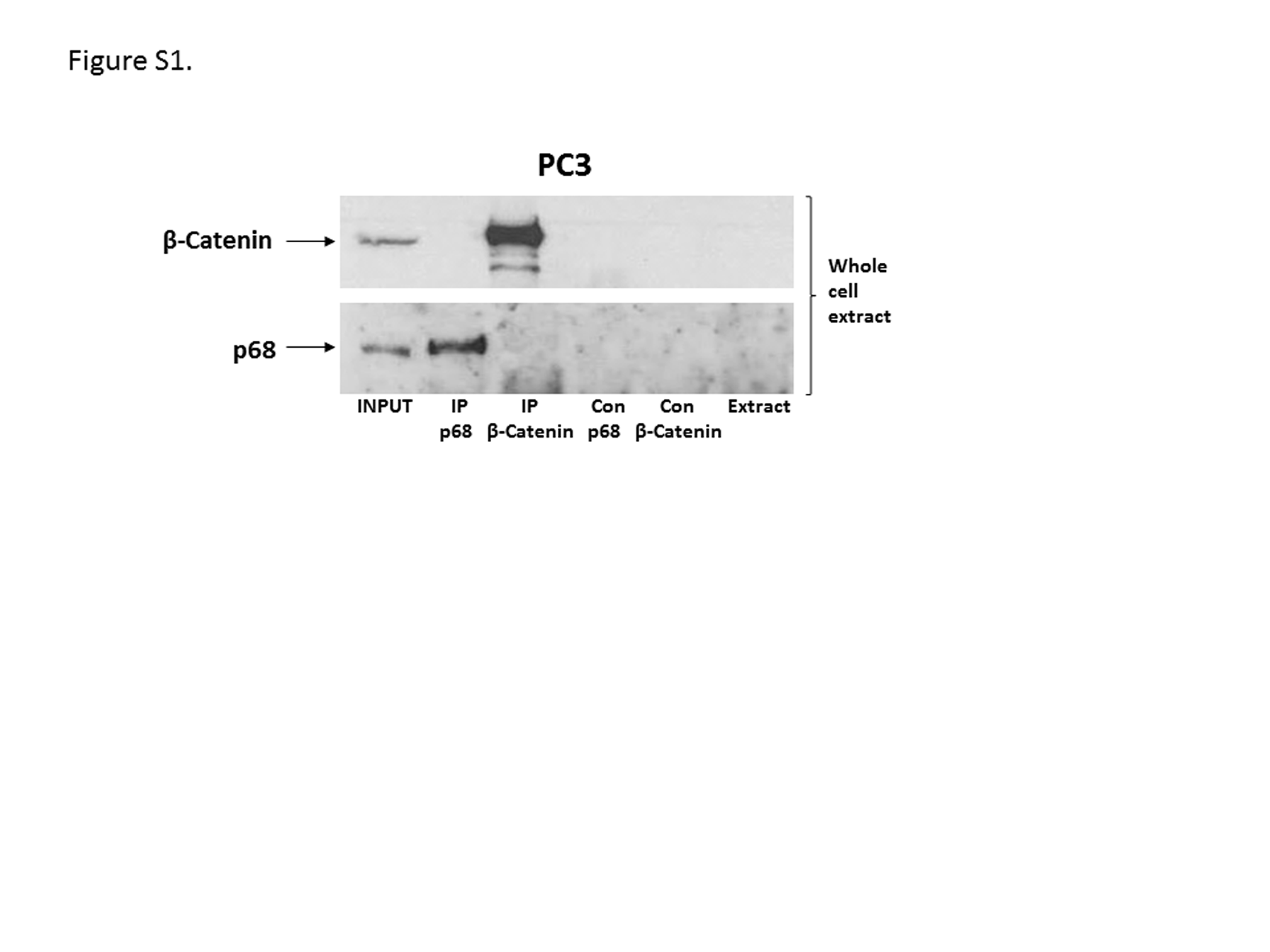

Supplement: Figure S1 — Immunoprecipitation of endogenous p68 and β-Catenin in an AR negative PC3 cell line do not interact. Cropped immuno-blot images of PC3 whole cell lysates, immunoprecipitated (IP) with either p68 or β-Catenin antibody and probed sequentially with β-Catenin and p68. Extract samples contain whole cell lysate and protein G sepharose with no antibody present, and Control (Con) samples contain antibody and protein G sepharose in extraction buffer only. (TIF) [file pone.0054150.s001.tif]

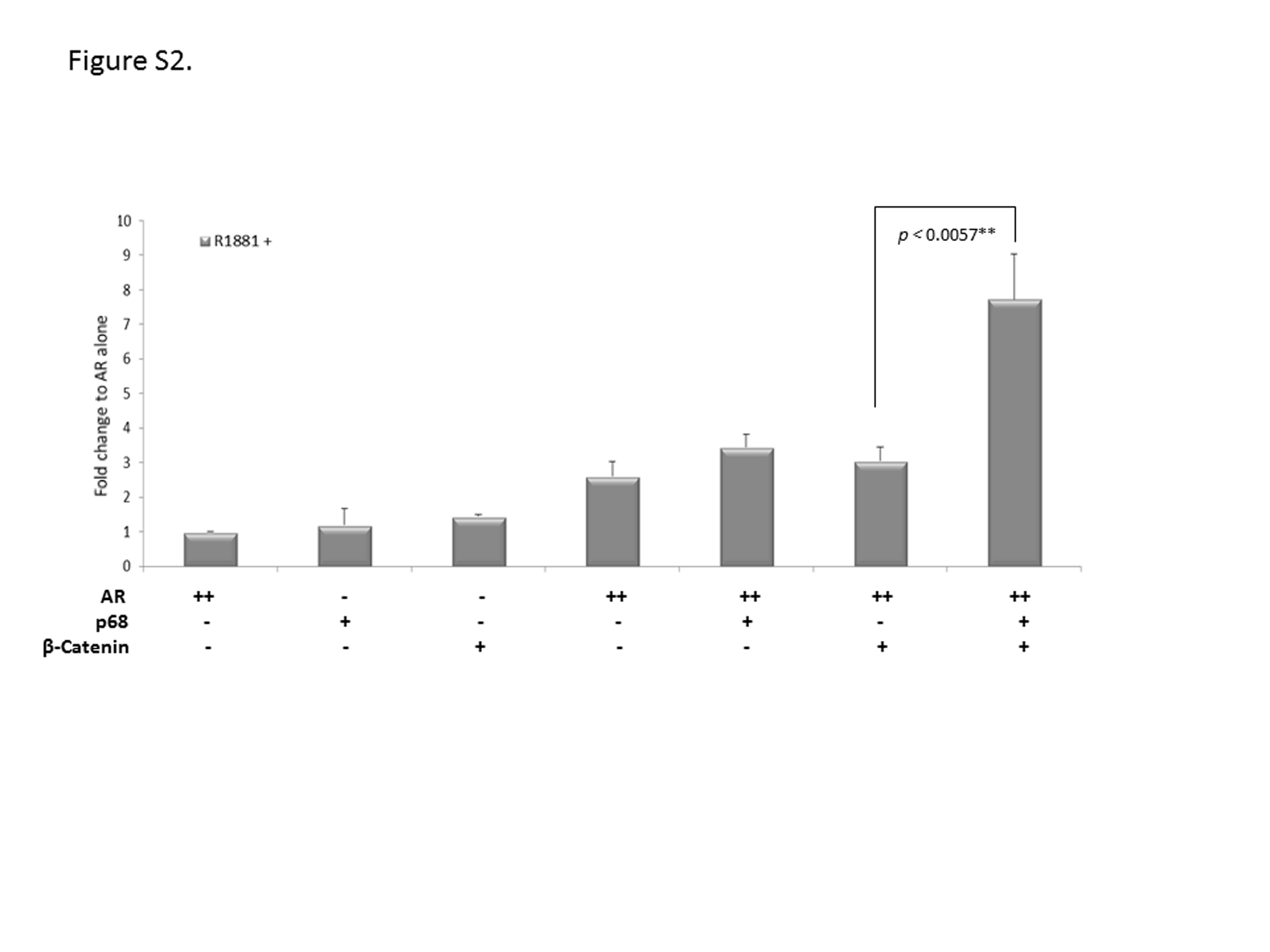

Supplement: Figure S2 — Over-expression of p68 additively enhances the activity of β-Catenin mediated AR transcription by an AR mediated PSA promoter luciferase reporter. COS-7 cells were transiently transfected in triplicate with 0.1 µg of p(PSA)Luc reporter, 0.1 µg pSG5-AR, 0.1 µg of pCMV-β-galactosidase and 0.05 µg of pcDNA3-p68 or pCS3+-Myc6-β-Catenin constructs (+10 nM R1881). Luciferase activity was corrected for the corresponding β-galactosidase activity to give relative activity. The range of plasmid levels (+ and ++) corresponds to 50 and 100ng respectively. Data is shown relative to AR activity alone (set as 1) and from at least three independent luciferase assay experiments (+/− SE). (TIF) [file pone.0054150.s002.tif]

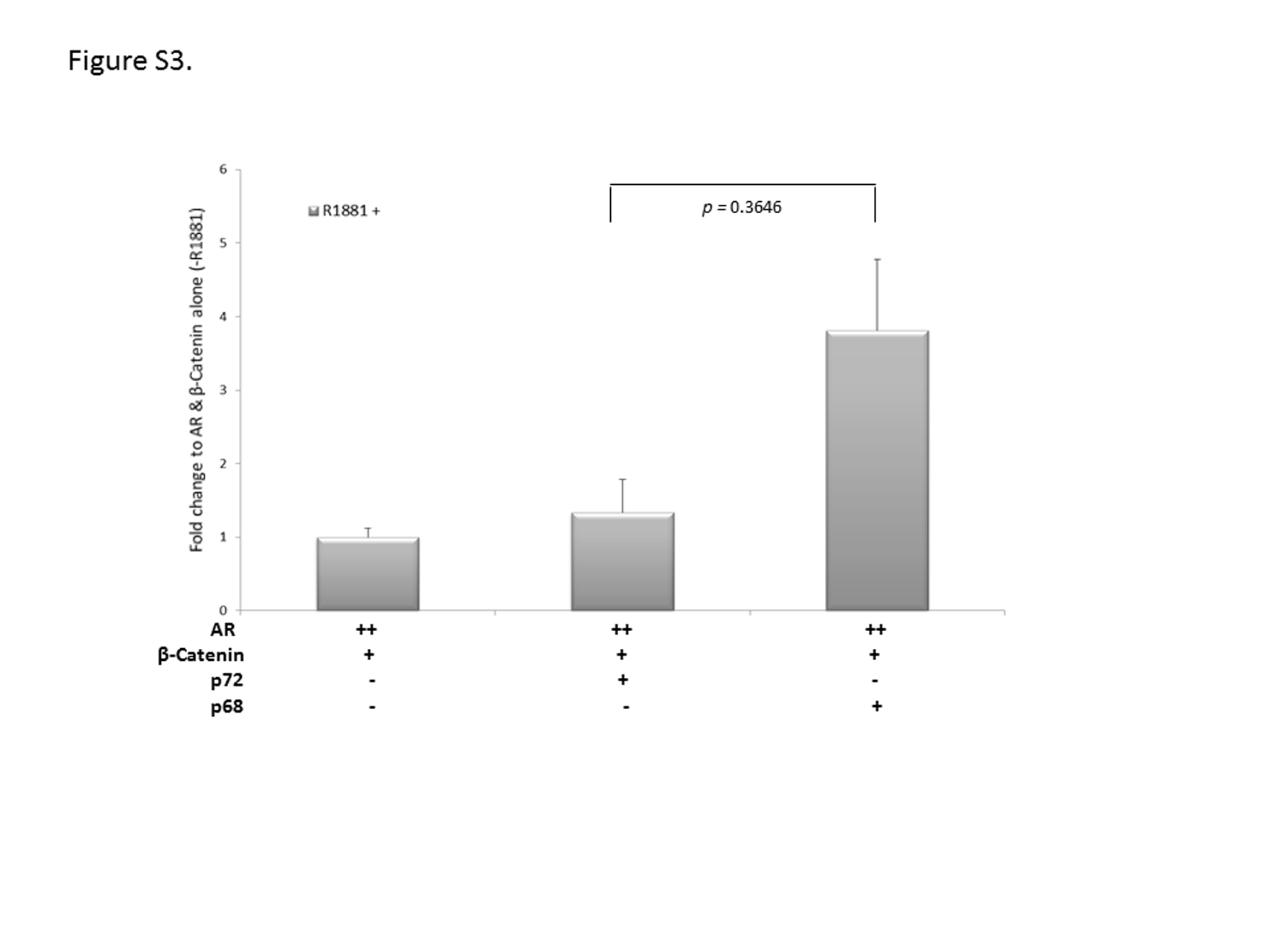

Supplement: Figure S3 — Over-expression of p72 does not enhance the activity of β-Catenin mediated AR transcription. COS-7 cells were transiently transfected in triplicate with 0.1 µg of p(ARE)3 luciferase reporter, 0.1 µg pcDNA3-AR, 0.1 µg of pCMV-β-galactosidase and 0.05 µg of pcDNA3-p72, p68 or pCS3+-Myc6-β-Catenin constructs (+10 nM R1881). Luciferase activity was corrected for the corresponding β-galactosidase activity to give a relative activity. The range of plasmid levels (+ and ++) corresponds to 50 and 100 ng respectively. Data is shown relative to AR and β-Catenin activity alone (set as 1) and from at least three independent luciferase assay experiments (+/− SE). (TIF) [file pone.0054150.s003.tif]

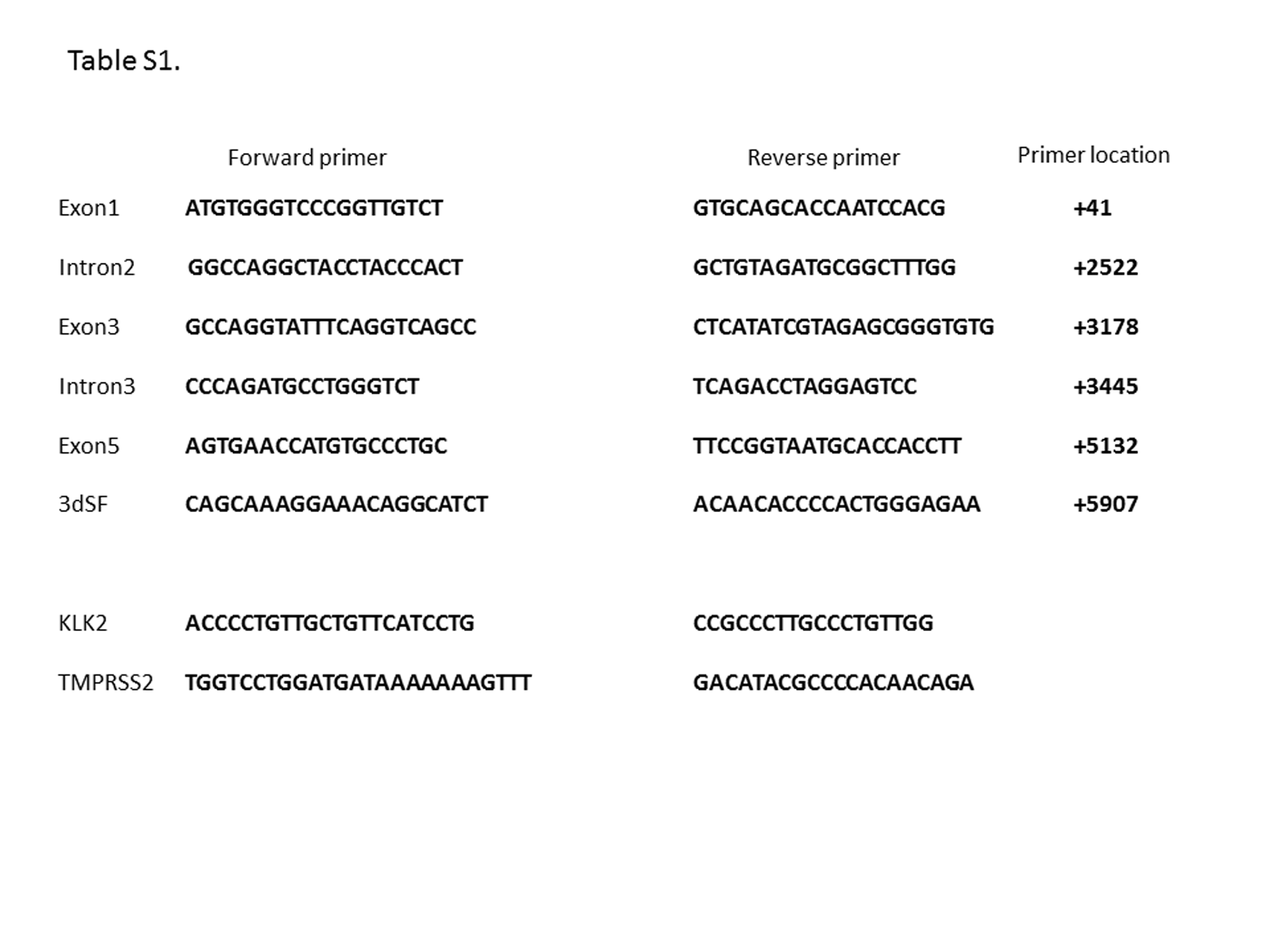

Supplement: Table S1 — Sequences for primers used in ChIP experiments and location of primers on the PSA gene. (TIF) [file pone.0054150.s004.tif]
